# Pain Characteristics and Associations with Quality of Life in Patients with Multiple Sclerosis in Lithuania

**DOI:** 10.3390/medicina56110596

**Published:** 2020-11-08

**Authors:** Greta Veličkaitė, Neringa Jucevičiūtė, Renata Balnytė, Ovidijus Laucius, Antanas Vaitkus

**Affiliations:** Department of Neurology, Medical Academy, Lithuanian University of Health Sciences, Eivenių street 2, LT-50009 Kaunas, Lithuania; greta.vel3@gmail.com (G.V.); neringa.juceviciute@gmail.com (N.J.); ovidijus.laucius@lsmu.lt (O.L.); Antanas.Vaitkus@lsmu.lt (A.V.)

**Keywords:** multiple sclerosis, pain, quality of life, anxiety, depression

## Abstract

*Background and objectives*: Even though pain in multiple sclerosis (MS) patients is common and possibly associated with reduced quality of life, its exact prevalence and characteristics remain vaguely understood. We aimed to estimate the true extent of pain and its associations with quality of life in Lithuanian MS patients and to compare this data with that of a control group. *Materials and Methods*: Data were collected prospectively at the Department of Neurology, Lithuanian University of Health Sciences Kaunas Clinics. A face-to-face structured interview and a questionnaire were used to collect demographic and clinical data of the MS (*n* = 120) and control (*n* = 120) groups. The Expanded Disability Status Scale (EDSS) was used to quantify disability in the MS group. Scores ≥4/10 in the Douleur Neuropathique 4 questionnaire were classified as neuropathic pain. Patients were evaluated using the anxiety and depression subsets of the Hospital Anxiety and Depression Scale (HADS-A and HADS-D), the physical and mental component subsets of the Short Form-12 questionnaire (PSC-12 and MSC-12). *Results*: The MS and control groups did not differ in pain prevalence (76.7% vs. 65.9%, *p* = 0.064) or intensity. Lhermitte sign, lower limb, and face pain were more common in the MS group, whereas subjects in the control group were more often affected by lower back, neck, and joint pain. Neuropathic pain and pain lasting longer than 2 years were more common among pain-affected MS patients than among controls. MS patients with pain had higher EDSS, HADS-D, and HADS-A and lower PSC-12 scores than those without pain; however, no difference was found regarding the duration of MS or age. Males with MS and pain had higher MSC-12 and HADS-D scores in comparison to the same subset of females. *Conclusions*: Pain affects approximately three out of four patients with MS in Lithuania and is negatively associated with the mental and physical aspects of quality of life.

## 1. Introduction

Pain is a common and significant symptom of multiple sclerosis (MS) that should not be dismissed, as it is rated as one of the highest priorities by individuals with MS, even surpassing functions such as speech or bowel and bladder control [1]. However, data on pain in MS are heterogeneous and prevalence values as low as 43% and as high as 86% have been reported [2,3]. Neuropathic pain is the type of pain most closely related to the pathology of MS and is found even in patients with early MS, with increasing frequency in later disease stages [4]; however, nociceptive pain also poses a marked problem as it is associated with musculoskeletal dysfunction that is a significant feature of MS. In addition, the presence of pain is likely associated with lower physical and mental quality of life as pain frequently accompanies depression and anxiety and vice versa [5]. Despite the prominent clinical importance of pain, its characteristics in MS remain only vaguely understood, and this lack of data limits the ability to provide patients with the best possible care. We aimed to estimate the true extent of pain and its associations with quality of life in MS patients in Lithuania as well as to compare these data with a control group.

## 2. Materials and Methods

Data were collected prospectively at the Department of Neurology, Lithuanian University of Health Sciences Kaunas Clinics. The MS group (*n* = 120) was composed of patients diagnosed with MS according to the 2010 and 2017 McDonald criteria [6,7]. Age- and gender-matched individuals who did not have MS or any neurodegenerative disease were included as control subjects (*n* = 120). All participants were >18 years old. Informed consent was obtained. The study was approved by the Lithuanian University of Health Sciences Bioethics Committee (Nr. BEC-LSMU®-40, 4 April 2019).

A face-to-face structured interview and a questionnaire were used to collect information on demographic data and also included questions about the course and clinical characteristics of MS and the frequency, duration, and management of pain. Disability in the MS group was quantified according to the Expanded Disability Status Scale (EDSS). The study subjects were presented a graphic outline of the body and were asked to mark the areas where they were experiencing pain. Patients were asked to separately report the intensity of the pain experienced at the time of assessment and the average intensity of pain experienced on the day before assessment. The Numeric Rating Scale (NRS-11) from 0 to 10 was used to evaluate the intensity of pain. The Douleur Neuropathique 4 (DN4) questionnaire was used to distinguish nociceptive and neuropathic pain: scores ≥4/10 were classified as neuropathic pain. The Hospital Anxiety and Depression Scale (HADS) was used to determine anxiety (HADS-A) and depression (HADS-D) levels: ≤7—normal; 8–10—borderline abnormal, possible mild anxiety or depression; ≥11—clinically significant anxiety or depression. The quality of life was assessed by using the Short Form-12 (SF-12) questionnaire and separately noting the scores of the physical component scale (PSC-12) and the mental component scale (MSC-12). 

Statistical analyses were performed using IBM SPSS Statistics for Windows, version 25.0 (IBM Corp., Armonk, NY, USA). Descriptive statistics were calculated. Data normality was assessed using histograms and the Shapiro–Wilk test. All variables were non-normally distributed; therefore, Mann–Whitney U test was used to evaluate the differences in continuous variables between the groups. Differences in categorical variables were assessed using the Fisher’s exact and chi-square tests. Data are presented as median (interquartile range) or number (percentage), unless indicated otherwise. Correlation was analyzed using Spearman’s rank correlation test. All *p* values are two-sided; *p* < 0.05 was regarded as statistically significant. 

## 3. Results

### 3.1. Associations between the Presence of Pain and Demographic and Clinical Characteristics

A total of 240 subjects were included in the study, their demographic and clinical data are presented in Table 1. The majority of MS patients (*n* = 119) were diagnosed with relapsing–remitting MS. Males had slightly higher EDSS scores than females (3.5 (2.4–4.5) vs. 2.5 (1.5–3.5), *p* = 0.021), no other differences between the genders in the demographic and clinical data were observed. The overall prevalence of pain differed neither between the MS and control groups nor between females and males in the MS or control groups. No differences were found between MS patients with pain and those who did not any pain regarding the duration of MS (9.0 (5.0–13.0) vs. 9.5 (5.0–13.0), years, *p* = 0.854) or age (44.0 (35.0–53.8) vs. 43.5 (28.5–49.8), years, *p* = 0.311).

### 3.2. Comparison of Pain Characteristics in the Multiple Sclerosis and Control Groups

Neuropathic pain and pain of more than 2 years’ duration were more common among MS patients who were experiencing pain compared to that among the control subjects affected by pain (*p* < 0.001), whereas no difference was found in the prevalence of constant pain and the intensity of pain. The percentage of participants who reported analgesic use did not differ between the groups. The comparison of pain characteristics between the MS and control groups is presented in Table 2. 

**Figure 1:**
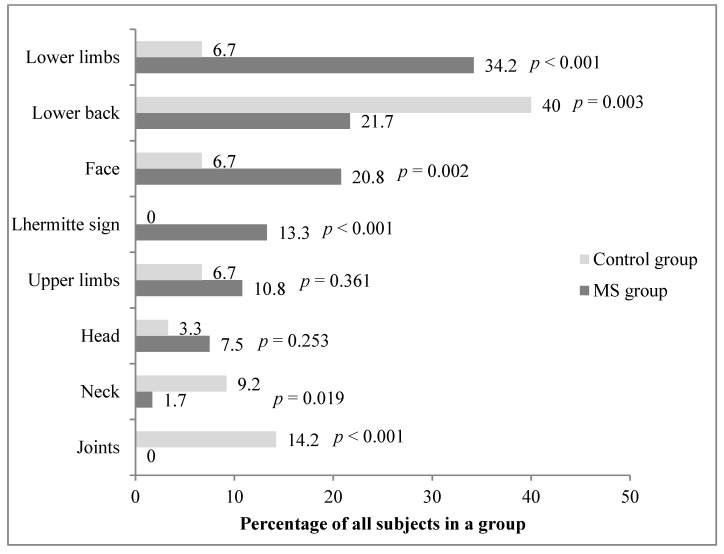
Pain location in the multiple sclerosis and control groups. Abbreviations: MS, multiple sclerosis.

Differences in pain location were also observed with Lhermitte sign, lower limb, and face pain being more common in the MS group, whereas subjects in the control group were more often affected by lower back, neck, and joint pain (*p* < 0.05) (Figure 1).

### 3.3. Associations between Pain and Both Phycical and Mental Aspects of Quality of Life

In the MS group, EDSS moderately correlated with HADS-D scores (*r* = 0.573, *p* < 0.001) and only weakly with HADS-A scores (*r* = 0.238, *p* = 0.009). Higher anxiety and depression levels and lower SF-12 scores were more common among MS patients than among controls regardless of their experienced pain. However, when comparing subjects without pain between the groups, only PSC-12 values were found to be lower in the MS group (data not shown). Therefore, it may be assumed that higher anxiety and depression levels in the MS group are caused by multiple factors, an important one being pain. Data on SF-12 and HADS scores with regard to the presence of pain are summarized in Table 3.

In the MS group, higher values of HADS-D scores and lower values of MSC-12 were found among males than among females (5.0 (4.0–10.0) vs. 4.0 (1.8–7.0), *p* = 0.011 and 40.6 (29.9–51.8) vs. 47.3 (37.6–57.5), *p* = 0.008) and the same was observed when comparing males with pain and MS to the same subset of females. No other gender-associated differences were detected (data not shown).

## 4. Discussion

Our study showed a 76.7% prevalence of pain among MS patients that is slightly higher than the prevalence of 62.8% that was reported in a systematic review and meta-analysis of pain in adults with MS [8]. We did not identify any difference in the duration of MS between patients with and without pain, and these results are consistent with the ones reported in certain studies [9,10,11] and contradictory with others [2]. A major source of these variabilities is the lack of consistency in inclusion criteria, such as differences in the proportion of males and females, EDSS scores, MS types as well as other clinical and demographic characteristics, which can lead to different results, e.g., those assessed in the outpatient setting are likely to report lower values of pain intensity and prevalence of pain than those managed in the inpatient setting.

A meta-analysis showed that nearly half of MS patients suffered from headache (42.5%) and that the most common pain syndromes were neuropathic extremity pain 26.6%, back pain 20.0%, and Lhermitte sign 16.6% [8]. Although quantitative results in studies of different patient populations and study designs cannot be directly compared, it is notable that some similarities in results have been obtained: a similar percentage of MS group patients in our study suffered from extremity pain (34.2% reported lower-extremity pain and 28.5% upper-extremity pain), back pain (21.7%), and Lhermitte sign (13.3%); however, our results did not confirm the high prevalence of headache as only 7.5% of MS patients reported having headaches. This number was much lower than the value we had expected. Given that the main focus of this study was on the overall prevalence and characteristics of pain without emphasis on headache, there is some likelihood that higher prevalence would have arisen if additional questions about headache had been included in the questionnaire.

A noteworthy observation to emerge from the data comparison was the detection of lower MSC-12 and HADS-D scores among males with MS and pain in comparison to those for females with MS and pain. A similar observation was reported in another study that showed better self-reported physical and mental health among females with MS [12]. A possible explanation of our results is that in our study males had higher EDSS scores, and we also found that EDSS scores moderately correlated with the HADS-D score and only weakly or insignificantly with other scores. Higher EDSS scores in males were not surprising, as male gender is known to be independently associated with faster disability accumulation in relapsing–remitting and secondary progressive MS, e.g., it has been calculated that it takes 8 years after the initial onset of symptoms for males to reach EDSS 3, while females reach this level of disability after 10 years [13].

We found that 52.5% of MS patients and 38.3% of controls (percentages of all subjects in each group) used pain medications and these numbers were much lower in comparison to the ones reported in a study that used information from the Swedish Prescribed Drugs Register to analyze the data of more than 3000 MS patients and 4000 controls [14]. The study reported that 79.5% of MS patients and 59.2% individuals in the non-MS cohort were prescribed pain medication [14]. These differences can be accounted for in part by contrasting pain management strategies in Europe that were shown in a large-scale study in 16 countries [15], for example, in Sweden only 30% of participants felt that at times their pain medicines were not adequate to control their pain in contrast to 74% of respondents in Denmark [15]. In addition, in 2015 the defined daily dose per 1000 inhabitants per day of non-opioid analgesics and antipyretics was 60% lower in Lithuania and 40% lower in Denmark in comparison with Sweden (71.0 and 107.4 vs. 179.1) [16]. There are several possible factors that could contribute to the differences in analgesic use: cultural influences, variations in the accessibility of non-pharmacological pain management programs; physicians’ prescribing habits; drug availability, pricing, and reimbursement; etc. 

Finally, the prevalence of pain in the control group (65.9%) was higher than we had expected. Due to a lack of data on this topic, the comparison of our results to the ones of other studies is complicated; however, a study in the USA that included more than 5000 White, non-Hispanic participants reported that the prevalence of pain in this population was 59.7% [17], which is only slightly lower than our reported result. It is noteworthy to emphasize that the prevalence of pain in individuals without MS would be assessed more accurately by random sampling in the wider population; therefore, the results of this study should be interpreted with caution.

Finally, a number of potential shortcomings need to be considered. The most important limitation lies in the fact that this study mainly relied on self-reported data obtained using questionnaires or self-assessment scales. In addition, this was a small-scale single-center study; therefore, the inclusion of participants was limited, and we were unable to gather data on pain and its characteristics in different MS types. This study was not specifically designed to accurately evaluate the prevalence and characteristics of headache; therefore, caution must be taken when interpreting the reported low prevalence of headache.

## 5. Conclusions

This was the first cross-sectional study to address the question of the prevalence and characteristics of pain in Lithuanian MS patients and to compare their data to controls. This work has revealed that there are no major differences in the prevalence or in the characteristics of pain in Lithuanian MS patients in comparison with the data reported by other authors. The present findings might suggest several courses of action in order to improve the well-being of MS patients. Firstly, physicians should not underestimate the prevalence of pain among those with MS as well as its effect on the mental and physical aspects of quality of life. In addition, given the low rate of pain medication use, it is possible that a significant percentage of MS patients do not receive adequate pain treatment. Pain in MS should be recognized as an important problem that requires the utmost diligence.

## Figures and Tables

**Table 1:** Demographic and clinical characteristics of the study population.

	MS Group (*n* = 120)	Control Group (*n* = 120)	*p* Value
Age, years	44.0 (31.3–52.0)	37.5 (28.0–53.0)	0.087
Females; males	86 (71.7%); 34 (28.3%)	85 (70.8%); 35 (29.2%)	1
MS duration, years	9.0 (5.0–13.0)	-	-
EDSS, points	2.5 (2.0–3.5)	-	-
Prevalence of pain	92 (76.7%)	79 (65.9%)	0.064

Abbreviations: EDSS, Expanded Disability Status Scale; MS, multiple sclerosis.

**Table 2:** Comparison of pain characteristics in the multiple sclerosis and control groups.

		Subjects Experiencing Pain (*n* = 171)		*p* Value
		MS Group (*n* = 92)	Control Group (*n* = 79)	
Age, years		44.0 (35.0–53.8)	41.0 (28.0–56.0)	0.509
Females; males		67 (72.8%); 25 (27.2%)	59 (74.7%); 20 (25.3%)	0.862
NRS-11, pain intensity	At the time of assessment	4.0 (2.0–5.0)	3.0 (2.0–5.0)	0.649
	On the day before assessment	4.0 (2.0–6.0)	4.0 (3.0–6.0)	0.799
Constant pain		24 (26.1%)	12 (15.2%)	0.093
Neuropathic pain		27 (29.3%)	3 (3.8%)	<0.001
Duration of pain	<6 months	18 (19.6%)	29 (36.7%)	0.014
	6 months–2 years	20 (21.7%)	20 (25.3%)	
	>2 years	54 (58.7%)	30 (38.0%)	
Use of analgesics		63 (68.5%)	46 (58.2%)	0.202

Abbreviations: MS, multiple sclerosis; NRS, Numeric Rating Scale.

**Table 3:** HADS and SF-12 scores in the MS and control groups.

		**MS Group**			**Control Group**			*p* Value *
		**Pain (*n* = 92)**	**No Pain (*n* = 28)**	***p* Value**	**Pain (*n* = 79)**	**No Pain (*n* = 41)**	***p* Value**	
HADS-A	Score, points	8.0 (4.0–11.0)	4.5 (1.3–8.0)	0.004	5.0 (3.0–8.0)	4.0 (3.0–6.5)	0.026	0.014
	Normal	43 (46.7%)	19 (67.9%)	0.130	57 (72.2%)	36 (87.8%)	0.148	0.003
	Possible mild anxiety	22 (23.9%)	5 (17.9%)		12 (15.2%)	3 (7.3%)		
	Clinically significant anxiety	27 (29.3%)	4 (14.3%)		10 (12.7%)	2 (4.9%)		
HADS-D	Score, points	5.0 (2.0–8.0)	3.5 (1.0–6.0)	0.034	3.0 (1.0–5.0)	2.0 (0.5–4.0)	0.032	0.001
	Normal	66 (71.7%)	24 (85.7%)	0.267	72 (91.1%)	40 (97.6%)	0.346	0.006
	Possible mild depression	14 (15.2%)	3 (10.7%)		4 (5.1%)	1 (2.4%)		
	Clinically significant depression	12 (13.0%)	1 (3.6%)		3 (3.8%)	0		
SF-12	PSC-12, points	33.6 (28.4–43.9)	48.1 (36.5–56.1)	<0.001	47.8 (38.4–52.1)	55.8 (50.9–56.8)	<0.001	<0.001
	MSC-12, points	43.4 (33.7–56.6)	52.1 (39.7–57.2)	0.081	50.9 (43.9–55.4)	49.5 (44.5–55.5)	0.821	0.005

Abbreviations: HADS-A, Hospital Anxiety and Depression Scale—Anxiety; HADS-D, Hospital Anxiety and Depression Scale—Depression; MS, multiple sclerosis; MSC-12, Mental Composite Scale-12; PSC-12, Physical Component Scale-12; SF-12, The Short Form-12 questionnaire; *p* value*—comparison between subjects experiencing pain in the multiple sclerosis and control groups.
